# Personalizing a Weight Loss Program Using Cognitive-Behavioral Phenotypes to Improve Engagement and Weight Loss in Adults With Overweight or Obesity: Quasi-Experimental Study

**DOI:** 10.2196/72645

**Published:** 2025-12-01

**Authors:** Joanna Szypula, Andreas Jarvstad, Lucy Amelia Jones, Katy Tapper

**Affiliations:** 1 School of Health and Psychological Science City St George's, University of London London United Kingdom; 2 Oviva London United Kingdom

**Keywords:** digital weight management program, health behavior, mobile applications, obesity, patient engagement, real-world evidence, weight loss intervention

## Abstract

**Background:**

Obesity affects more than one-quarter of adults in the United Kingdom and is a leading cause of preventable disease and health care costs. Digital behavior change programs can provide scalable weight management support, but maintaining engagement is challenging, and engagement is strongly associated with weight loss success. Tailoring interventions to cognitive-behavioral phenotypes, distinct patterns of thinking and behavior, offers one strategy to improve adherence. Although such approaches show promise in controlled settings, evidence from real-world digital programs is limited.

**Objective:**

This study evaluated whether phenotype-tailored weekly advice improved engagement and weight loss in a national digital weight management program. Secondary aims were to assess correlations between advice use and outcomes, explore moderation by socioeconomic status, and capture participants’ perceptions of the advice.

**Methods:**

We conducted a quasi-experimental study among UK adults enrolled in a free 12-week program commissioned by the National Health Service. Eligible participants were aged 18-80 years with a BMI greater than 25 kg/m². The phenotype group (n=148; mean age 48 years; 127/148, 86% female; mean BMI 39 kg/m²) completed a 17-item questionnaire, were classified into one of 4 phenotypes, and received weekly tailored advice for 7 weeks. Comparators included a historical cohort enrolled 1 year earlier without phenotype advice (n=241; mean age 44 years; 171/241, 71% female) and nonresponders who did not complete the questionnaire (n=394; mean age 44 years; 299/394, 76% female). Primary outcomes were program engagement (any in-app activity such as meal logging, activity tracking, content reading, or coach messaging) and self-reported weight.

**Results:**

The phenotype group recorded a mean of 257 (SD 232) engagements over 7 weeks, significantly higher than both the historical cohort (mean 159, SD 187; *P*<.001) and nonresponders (mean 135, SD 198; *P*<.001), representing 62%-90% greater activity. All engagement types were significantly elevated (*P*<.001 for all). Mean weight loss was −2.23 kg (SD 7.97) in the phenotype group, compared with −1.60 kg (SD 5.39; *P*=.29) in the historical cohort and −0.69 kg (SD 13.23; *P*=.23) in nonresponders. The number of phenotype-specific advice documents opened correlated with engagement (*r*=0.48; *P*<.001) but not with weight loss (*P*=.42). Socioeconomic status did not moderate outcomes. Posttrial interviews (n=16) provided mixed feedback: many participants described the advice as clear, relevant, and motivating, whereas others considered it too general or poorly matched.

**Conclusions:**

Phenotype-tailored weekly advice was associated with substantially higher engagement in a real-world digital program, although short-term weight differences were not statistically significant. While limited by a nonrandomized design, short follow-up, and reliance on self-reported weight, this study suggests phenotype-based tailoring may be a scalable strategy to strengthen adherence in digital weight loss interventions. Larger randomized trials with longer follow-up are warranted to determine whether increased engagement translates into clinically meaningful weight loss.

## Introduction

Global obesity rates have doubled since 1990 [1], with approximately 1 in 4 adults in the United Kingdom now living with the condition [2]. Although early interventions focused on telling individuals to “eat less and move more,” research over the past 2 decades has highlighted obesity as a complex, multifactorial issue requiring multicomponent, evidence-based treatment [3].

Many weight loss interventions are generic, offering identical advice to all participants [4,5]. This may partly explain the wide variability in individual responses, even to intensive treatments such as bariatric surgery [6]. Recent efforts have focused on personalizing interventions, often through genetic, anthropometric, or microbiota-based approaches [7-9]. There is growing evidence that personalized nutrition and exercise advice can enhance outcomes [8-10], but comparatively little attention has been paid to tailoring weight loss interventions based on cognitive-behavioral characteristics. Some research has used tools such as the Three-Factor Eating Questionnaire, Dutch Eating Behavior Questionnaire, or 6-Factor Questionnaire to generate behavioral profiles [11-13], but the effects of tailoring advice based on these profiles remain largely untested.

Digital, app-based weight loss interventions are increasingly popular because of their scalability, accessibility, and low cost [14]. They can be as effective as, or more effective than, in-person programs [15-17] and allow for large-scale, real-world data collection to assess adherence and outcomes [18]. Individual differences, such as personality, may influence in-app engagement and outcomes in digital weight management programs [19]. Tailoring interventions has been shown to increase engagement, which may lead to better outcomes [20-22], possibly because personalized advice feels more relevant and persuasive [23-26]. Improving engagement is particularly important in digital settings, where high attrition can limit effectiveness [26].

In-app engagement is commonly used as a proxy for adherence, with more frequent interaction indicating greater engagement [20]. A recent analysis by Lehmann et al [18] found a significant relationship between Oviva (Oviva AG) app engagement and weight loss at 3 months across all countries analyzed but not specifically within the United Kingdom. This nonsignificant relationship in the United Kingdom may be explained by the fact that these data came from more complex patients living with obesity, who often face greater challenges, such as previous unsuccessful weight loss attempts, reliance on prescribed treatments (eg, glucagonlike peptide-1 receptor agonists or total dietary replacement), higher emotional eating, loss of control, significant comorbidities, and psychological barriers [27,28]. These factors could account for the weaker association between app engagement and weight loss in this group. Nevertheless, the broader international findings support the interpretation that app engagement reflects meaningful behavioral activity. App features prompt users to engage in evidence-based techniques such as self-monitoring, reflection, and problem-solving, all known to facilitate weight loss [29]. Therefore, increases in app engagement are likely to reflect active participation in self-management strategies, which may contribute to improved weight outcomes.

For this study, we collaborated with Oviva, a provider of digital weight loss services commissioned by the United Kingdom’s National Health Service (NHS). In a previous study, we developed a 17-item questionnaire to assign users to 1 of 4 cognitive-behavioral phenotypes [30]. In this study, we aimed to test whether receiving phenotype-tailored advice would improve app engagement and weight loss in a real-world setting. Using a quasi-experimental design, patients enrolled in the Oviva program were invited by email to complete a “quiz” that provided personalized advice. Those who responded received weekly, tailored emails for 7 weeks. Two comparison groups were included: a historical cohort of patients enrolled a year earlier and a group of nonresponders who received the invitation but did not click the “quiz” link.

As socioeconomic status is known to be associated with obesity prevalence [31-35], but its role in digital program outcomes remains unclear, we additionally explored this in this study. Some evidence suggests that eHealth interventions can support weight loss in low-income adults [36], whereas others report no effect [37-39]. However, many studies may be underpowered to detect interactions or may not recruit representative samples from lower socioeconomic groups [40-44].

We hypothesized that patients receiving phenotype-tailored advice would show a greater number of in-app engagements and would lose more weight than the comparison groups. We also predicted that these effects would be moderated by socioeconomic status (using the Index of Multiple Deprivation [IMD] as a proxy), with less benefit among more deprived participants. Finally, we expected greater app engagement and weight loss among those who accessed more of their phenotype-matched content.

## Methods

### Ethical Considerations

Ethical approval for this study was granted by the City, University of London Psychology Department Research Ethics Committee (ETH2324-1944; amendments ETH2324-2135 and ETH2324-2449). Participants did not sign a separate consent form because patients enrolling in the Oviva program provide broad consent to be included in trials and for their anonymized data to be used for research, including secondary analyses. Eligible patients received an email inviting them to complete the phenotype questionnaire, with a clear notice that accessing the survey would enroll them in a 7-week automated email sequence. Participation was voluntary, and recipients could unsubscribe at any time. Historical comparison data were fully anonymized and covered under the original consent. Email addresses were used only to match individuals to anonymized patient user IDs, after which all identifiers were replaced. App engagement data were analyzed in anonymized form. Only authorized internal teams accessed identifiable data for email delivery and reporting, and no personal information was included in the research data set. Participants were not financially compensated but received weekly educational materials as part of the intervention.

### Participants

Participants were recruited from Oviva, an NHS-contracted weight loss service provider that is free for patients. They were sent an invitation email if they had started their weight management program with Oviva within the 3-week recruitment period in May 2024. The weight management program used for recruitment is a 12-week digital behavioral and lifestyle intervention offered to NHS-referred patients. It includes support from specialist weight management dietitians and health coaches. Participants gain access to the Oviva app, a class IIa medical device that features lessons on weight management and healthy lifestyle, goal setting with reminders, behavior tracking (eg, food, fluid intake, and physical activity), and communication with their coach to monitor progress. Any activity on the app, such as logging, messaging the coach, or reading a lesson, was recorded as an “engagement.” As this was a pragmatic, quasi-experimental study using convenience sampling over a fixed 3-week recruitment period, enrollment was determined by natural sign-up rates.

### Program Eligibility

To join the weight management program at Oviva, patients were required to meet the following eligibility criteria: be aged 18-80 years; reside in, or be registered with, a general practitioner practice in England; have a BMI ≥30 kg/m² (or ≥27.5 kg/m² for individuals of Black African, African-Caribbean, or Asian origin); have a diagnosis of diabetes or hypertension; own a smartphone; and be able to speak English. Patients were ineligible if they had severe or moderate frailty as recorded on a frailty register, were pregnant, had been diagnosed with an eating disorder, had a significant unmanaged comorbidity (eg, chronic obstructive pulmonary disease not on medication), or had undergone bariatric surgery in the past 2 years.

### Design

This study used a quasi-experimental design with posttest nonequivalent comparison groups. All Oviva weight management patients who started their program within the recruitment window were invited to complete the phenotypes quiz. Those who clicked the link and completed the questionnaire were assigned to the phenotypes group. Two comparison groups were included: (1) patients who enrolled in the same program 12 months before recruitment (historical cohort) and (2) patients who were invited to complete the questionnaire but did not click the link (nonresponders). The historical cohort was randomly selected by the Oviva analytics team, and the researchers had no access to, or influence over, which patients were included. The historical cohort served as a comparable reference group, as no substantial modifications had been made to the app or program structure since their participation. Although minor updates and iterative improvements occurred, these were not expected to meaningfully impact engagement or outcomes.

The dependent variables were weekly app engagements and weight loss over a 7-week period. Any of the following activities counted as an app engagement: tracking an event (eg, meal, fluid, waist circumference, mood), sending a chat message to a coach, opening a lesson page (each page viewed increased the engagement count), or opening a weekly article.

### Materials and Measures

#### Demographics

Participant demographics were provided by Oviva’s analytics team, typically sourced from general practitioner practices, with weights self-reported. Socioeconomic status was indicated by IMD decile, a UK measure that estimates poverty based on geographical location and considers factors such as income, employment, crime, and education (higher IMD scores indicate lower deprivation [45]).

#### Phenotypes Questionnaire

The phenotypes questionnaire, or “quiz,” is a 17-item questionnaire developed in our earlier study to match patients to 1 of 4 profiles [30]. Briefly, we identified 5 cognitive and behavioral constructs that (1) could affect weight or weight loss efforts, (2) have an existing validated measure, and (3) are potentially modifiable. The 5 constructs were maladaptive eating, hedonic overeating, reward reactivity, self-regulation, and psychological avoidance (Multimedia Appendix 1 contains the full questionnaire). For further methodological details, please consult the development paper (currently under review) [46].

The quiz was created in Qualtrics (Qualtrics International Inc), with each item rated on a scale from 1 (strongly disagree) to 5 (strongly agree). Participants were matched to a phenotype by calculating a percentage fit for each profile and assigning them to the one with the highest fit. They were shown their profile match along with a short, 3- to 4-bullet point description of their phenotype. Participants could either continue with their matched phenotype or review all profile descriptions and choose the one that resonated most with them. If 2 or more fit scores were equal, participants were shown all profile descriptions and asked to select one.

#### The Phenotypes

There were 4 phenotypes a participant could be matched with. The profiles were named after colors to keep the assignment emotionally neutral, and each was paired with a spice name to create a distinctive cue for participants, helping them remember their profile name over the following weeks. “Red Chilli” was characterized by high maladaptive and hedonic eating, low self-regulation, and high psychological avoidance. “Yellow Saffron” was based on high hedonic eating and reward reactivity and low maladaptive eating. “Purple Lavender” represented low self-regulation and high psychological avoidance, as well as low maladaptive and hedonic eating. Lastly, “Green Sage” reflected low maladaptive and hedonic eating, high self-regulation, and low psychological avoidance.

The 4 cognitive-behavioral phenotypes are intended as practical, nonexclusive groupings that highlight dominant behavioral tendencies rather than rigid or mutually exclusive categories. Given the complexity of eating behavior, some overlap in traits and relevance of advice is expected. The profiles aim to guide individuals in identifying and addressing key obstacles to weight loss while acknowledging that multiple factors may coexist.

#### Phenotype Content

Participants received weekly emails with advice tailored to their phenotype. The first document provided an overview and initial content, followed by weekly strategies addressing specific challenges (eg, overeating). Each document provided evidence-based advice and a short exercise to help participants put this knowledge into practice.

The Red Chilli profile focused on reducing restriction and maladaptive eating using acceptance and commitment therapy, mindfulness, cognitive defusion, and self-compassion. The Yellow Saffron profile addressed hedonic overeating with smaller portions of enjoyable foods, sensory eating techniques, craving management, and environmental architecture for healthier choices. The Purple Lavender profile improved goal setting and adherence by engaging with health goals daily, starting with small behaviors, and using routines, cognitive architecture, and defusion for challenging thoughts. Finally, the Green Sage profile enhanced diet quality and physical activity by adding vegetables, choosing foods with longer oral processing times, applying flexible eating (80% nutritious, 20% less nutritious), and cognitive architecture for activity goals.

#### Posttrial Feedback

After receiving all content emails, participants were sent a final email requesting feedback through a Qualtrics questionnaire, which included 11 Likert scale questions (1=strongly disagree to 5=strongly agree) assessing the helpfulness, effectiveness, and clarity of the advice. Participants were also given 4 optional open-ended questions to comment on the quiz, useful aspects, and suggestions for improving their profile or the advice.

#### Procedure

Participants were sent an email inviting them to take part in the phenotypes quiz. Those who completed the quiz were automatically matched to a profile and asked to read through its description, based on which they could decide to accept their match or change it. Once their response was submitted, they received an automatic email reminding them which profile they were matched with and were added to a 7-week long campaign.

Participants received the first email with tailored advice 1 day after they took the quiz. This email contained links to 4 documents (1 for each phenotype), with participants instructed to open the one matching their profile. Six more weekly emails followed, each containing 4 profile-specific documents. Thus, throughout the trial, participants received links to 28 documents but were instructed to only open 7 of them. The final email contained a link to the posttrial feedback questionnaire. The core program and care provided by dietitians and health coaches remained unchanged.

Although the tier 2 program lasts 12 weeks, the phenotype-specific advice was delivered over a 7-week period due to project timelines, which required all data collection and analysis to be completed within a fixed timeframe.

### Analysis Plan

The main analyses pertained to the effect of receiving phenotype-related advice on the average number of app engagements and weight loss over a 7-week period. Hierarchical regressions were conducted to compare the average number of app engagements between the phenotype group, and the historical cohort group, and the nonresponder group. We tested whether IMD moderated this relationship by including it as step 2 of the regression and the interaction term as step 3. The same approach was used to analyze the effect of condition on weight loss.

We further carried out equivalent Bayesian analyses using STAN (Stan Development Team) using *stan_glm* in rstanarm version 2.32.1 (Stan Development Team), brms version 2.22.0 [47], R version 4.4.1 (R Foundation for Statistical Computing), and RStudio 2024.12 (Posit Software, PBC). We fit 4 models for each dependent variable (DV): (1) DV ~ 1 (intercept only), (2) DV ~ group, (3) DV ~ group + IMD, and (4) DV ~ group * IMD. The engagement variable was square root–transformed, and the IMD variable was standardized prior to fitting. The weight loss variable contained outliers and a preponderance of zeros. To maintain parity with the standard regression analyses, we did not further process these data in any way. We used weakly informative robust *t*-distribution priors (*df*=3; mean 0, SD 10), and a Gaussian link function. We ran 8 chains, each with 1000 discarded burn-in samples and 5000 retained samples, for a total of 20,000 retained samples per model. We verified model convergence using standard criteria (eg, R̂ ~ 1) and visually inspected chains for convergence. For each model, we computed the log marginal likelihood and computed Bayes Factors (BFs) first for target models against the null intercept-only model. For any models which provided BF in favor of the target models over the intercept-only model (ie, BF>3), we additionally computed pairwise BFs to establish which of the surviving models best explain the data.

Change in the average number of engagements on a week-by-week basis was analyzed using a repeated-measures ANOVA.

Qualitative feedback from open-ended survey responses and semistructured interviews was analyzed using a descriptive thematic approach. Responses were reviewed inductively to identify recurring sentiments and contrasting views across participants. Illustrative quotes were selected to reflect the diversity of opinions, and analysis focused on summarizing key perceived benefits, limitations, and personal relevance of the intervention.

The study protocol was uploaded to the Open Science Framework before data analysis, and the analysis plan was preregistered before obtaining engagement and weight loss data from Oviva [48]. Data were analyzed using SPSS (version 28; IBM Corp).

## Results

### Participant Characteristics and Study Flow

**Figure 1:**
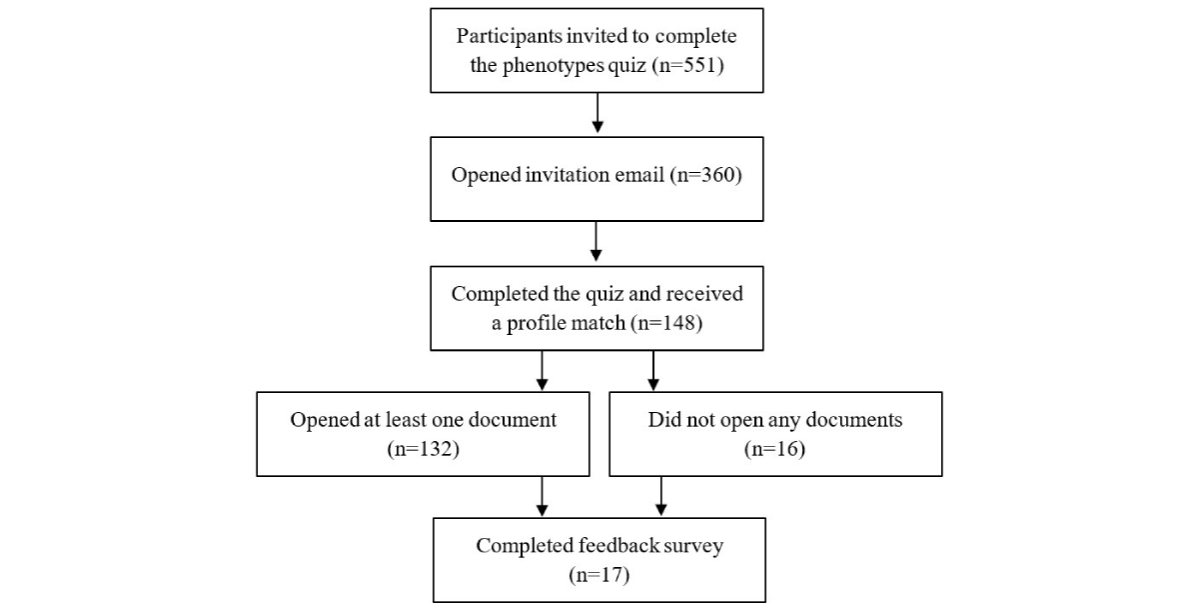
Flow of participants in a quasi-experimental study evaluating the effect of receiving phenotype-matched weight loss advice on app engagement and weight loss among patients actively participating in a UK weight loss program, recruited in May 2024.

Figure 1 shows the flow of participants through the study. Participant demographics were reasonably well matched across the 3 groups (Table 1), although participants in the phenotype group were slightly older, more likely to be female, and had a higher average IMD score than those in the comparison groups.

**Table 1:** Demographic characteristics of participants in the phenotype group and two comparison groups (historical cohort and nonresponders). Scores range from 1-10; lower values indicate higher relative deprivation.

Characteristic			Phenotypes group (n=148)		Historical cohort (n=241)		Nonresponders (n=394)
Age (years), mean (SD)			48 (12)		44 (10)		44 (11)
Female sex, n (%)			127 (86)		171 (71)		394 (76)
Starting weight (kg), mean (SD)			108 (19.7)		111 (24.1)		109 (25.0)
Starting BMI (kg/m^2^), mean (SD)			39 (6)		39 (8)		39 (8)
**Ethnicity, n (%)**							
	White	127 (86)		166 (69)		280 (71)	
	Black	8 (5)		39 (16)		41 (10)	
	Asian	7 (5)		24 (10)		33 (8)	
	Mixed	3 (2)		2 (1)		19 (5)	
	Other	2 (1)		7 (3)		12 (3)	
	Refused response	0 (0)		2 (1)		6 (2)	
	Missing data	1 (1)		1 (1)		3 (1)	
**Index of Multiple Deprivation (IMD)**							
	Mean (SD)	6 (3)		5 (3)		5 (3)	
	Median (IQR)	6 (8-3)		3 (7-1)		5 (7-3)	

### Phenotype Assignment

**Figure 2:**
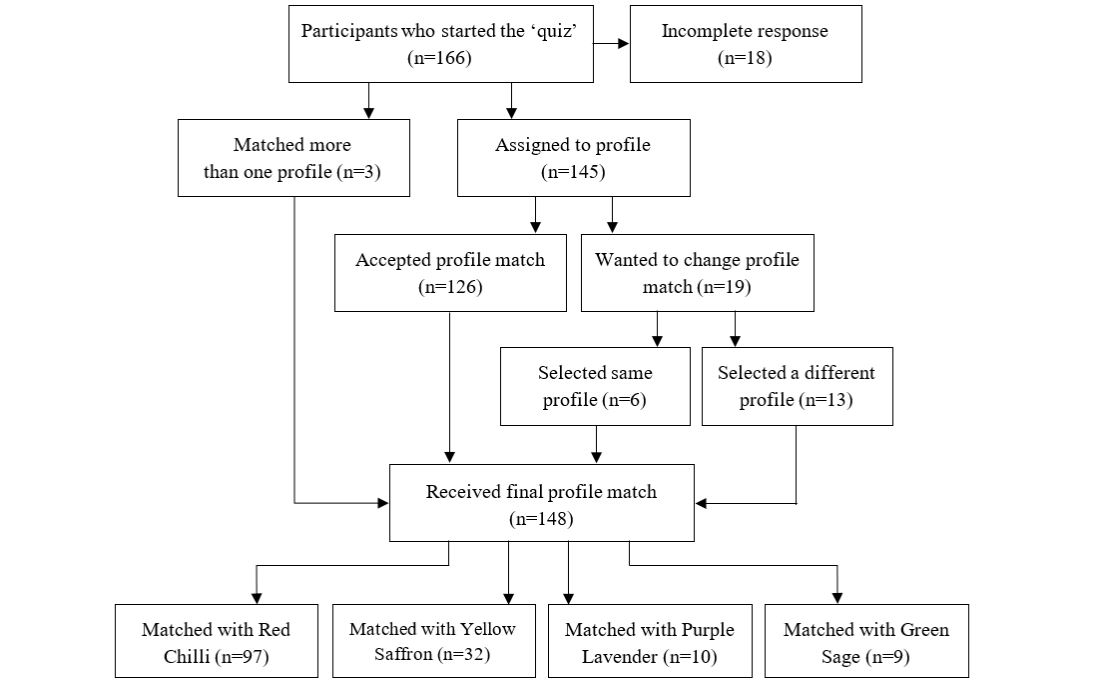
Flow of participants in the phenotype group from starting the phenotype quiz to receiving a final profile match, which determined the tailored weight loss advice they received during the trial.

Figure 2 shows how phenotype-group participants were assigned to their final profile. A total of 18 patients who received an invitation to the quiz clicked on the link but did not complete the questionnaire; therefore, they received the weekly emails but were unaware which links they should click on. Data from these participants were excluded from all analyses.

### Phenotype Fidelity

Participants were asked to open only documents for the profile they were matched with (eg, those matched with Red Chilli should open Red Chilli documents in weeks 1-7). However, since documents for all profiles were sent each week, it was possible for participants to open advice for multiple profiles. Fidelity was defined as opening at least 1 document tailored to the profile the participant was matched with and not opening more than 1 document from a different profile. The fidelity rate was 73.6% (109/148) throughout the trial. Among the remaining participants, 4.1% (6/148) followed advice for a different profile (opened 3 or more documents for a different profile and no more than 1 document for their original profile), 10.8% (16/148) opened no documents, and 11.5% (17/148) showed a different click pattern.

### Main Analyses

#### Effect of Condition on Total Engagements

The average total number of engagements over a 7-week period was higher in the phenotype group than in the historical cohort (Table 2). A hierarchical linear regression was conducted to compare the average number of engagements between the two groups, and to examine whether this effect was moderated by IMD. Condition was entered at step 1, IMD at step 2, and the interaction term at step 3. The analysis revealed that participants in the phenotype group generated significantly more engagements than those in the historical cohort (b=−93.61, SE B=22.39, β=−0.22; *P<*.001), but the interaction was not significant (b=−4.44, SE B=7.54, β=−0.12; *P*=.56). An equivalent Bayesian analysis confirmed that a model including only an intercept term (B=11.91, 95% CI 11.05-12.74) and the main effect of phenotype group (B=1.89, 95% CI 1.05-2.74) was superior to the model that also included a main effect of IMD (BF=144) as well as the interaction model (BF=1429). All the models were superior to the baseline intercept-only model (all BFs>14). Independent *t* tests (2-tailed) showed that all engagement subtypes (tracking activity, coach messaging, pages of educational modules viewed, and articles read) were significantly higher in the phenotype group than in the historical cohort group, suggesting that the overall increase in engagement was not driven by any single type of app interaction (*P*<.001 for all; Multimedia Appendix 1 contains all analyses).

**Table 2:** Mean number of app engagements and mean weight loss in the phenotype group and two comparison groups (historical cohort and nonresponders).

Group	Total app engagement count, mean (SD)	Weight loss (kg), mean (SD)
Phenotypes group (n=148)	257 (232)	2.23 (7.97)
Historical cohort (n=241)	159 (187)	1.60 (5.39)
Nonresponders (n=394)	135 (198)	0.69 (13.23)

#### Effect of Condition on Weight Loss

Average weight loss over a 7-week period was compared between the phenotype group and the historical cohort (Table 2). Another hierarchical regression was conducted to test the effect of condition on weight loss and whether IMD moderated this relationship. Condition was entered at step 1, IMD at step 2, and the interaction term at step 3. No significant differences in weight loss were found between the two groups (b=0.85, SE B=0.80, β=0.06; *P*=.29), and the interaction was not significant (b=−0.26, SE B=0.27, β=−0.22; *P*=.33). The equivalent Bayesian analysis showed that a baseline intercept model was substantially better than all the main effect and interaction models (all BFs≥567), indicating that neither group, IMD, nor their interaction explained variation in weight loss.

#### Relationship Between Documents Opened and Primary Outcomes

Among the phenotype cohort there was a significant positive correlation between the number of correct documents opened (ie, documents that matched the phenotype the participant was assigned to) and total engagements (*r*_146_=.481; *P<*.001). There was no relationship between the number of correct documents opened and weight loss (*r*_119_=−.074; *P*=.42).

#### Comparison With Nonresponders

An independent-groups *t* test (2-tailed) revealed participants in the phenotypes group generated significantly more engagements over the 7-week period than nonresponders (equal variances not assumed; Levene Test *F*_1,540_=12.98; *P<*.001; t_232.25_=5.68; *P<*.001; Table 2). Weight loss over the 7-week period was higher among the phenotype group compared with nonresponders, but this difference did not reach statistical significance (t_445_=−1.21; *P*=.23; Table 2).

#### BMI and Body Mass Percentage Reduction

On average, participants in the phenotype group reduced their BMI by 0.79 kg/m^2^ (SD 2.90), compared with a reduction of 0.56 kg/m^2^ (SD 1.94) in the historical cohort over the 7-week period. This translated into a reduction of 1.93% (SD 7.24) in body mass in the phenotype group and a 1.27% (SD 4.14) reduction in body mass in the historical cohort. Nonresponders reduced their BMI by 0.11 kg/m^2^ (SD 5.01) and their body mass by 0.09% (SD 13.01).

### Exploratory Analyses

**Figure 3:**
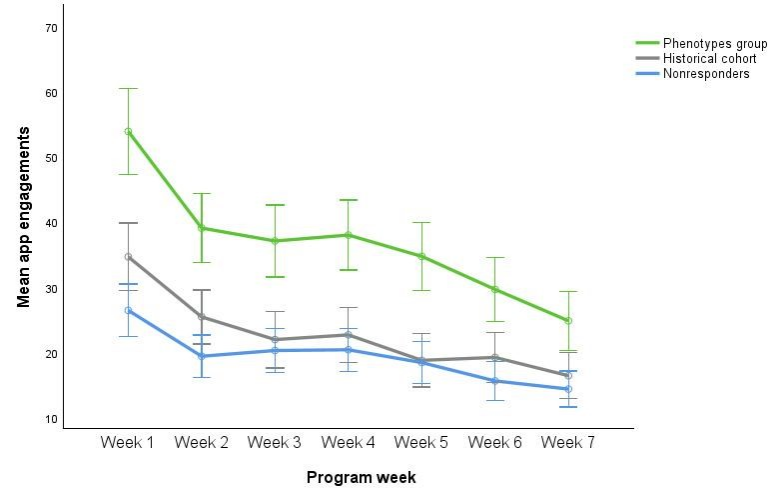
Mean weekly app engagements in the phenotype group (n=148) and the two comparison groups (historical cohort, n=241; nonresponders, n=394). Error bars denote 95% CIs.

A repeated-measures ANOVA was conducted to assess week-by-week change in app engagements and the interactions between the phenotype group, the historical cohort, and the nonresponders. As Mauchly test of sphericity was significant (*χ*^2^_20_=715.1; *P*<.001; Mauchly W=0.40; Greenhouse-Geisser correction ε=.720), the Greenhouse-Geisser correction was applied. There was a significant effect of week number on app engagement (*F*_4.32,3367.68_=81.85; *P<*.001), and the interaction with condition was significant (*F*_8.64,3367.68=_5.57; *P<*.001; Figure 3).

### Posttrial Ratings

Posttrial feedback was provided by 17 participants (12.2%); however, 1 participant did not open any documents with phenotype-tailored advice, and their feedback was therefore excluded from the analysis. Overall, feedback on the phenotype-tailored advice was positive (Table 3), as participants felt the advice they received was helpful and easy to understand. Participants also indicated that they were willing to use the advice in the future.

**Table 3:** Posttrial participant ratings of phenotype-tailored advice (n=16), including perceived helpfulness, clarity, personalization, and intention to use the advice, scored on a 1-5 scale, with higher ratings indicating greater endorsement.

Statement	Rating (SD)
The advice I received was helpful	3.75 (1.13)
The advice helped me make positive changes to my lifestyle	3.63 (1.26)
The advice helped me to lose weight	3.25 (1.24)
The length of the advice was just right (not too short and not too long)	3.50 (1.37)
The advice was clear and easy to understand	4.06 (1.00)
I learned something new by reading the advice in the resources	3.44 (1.31)
The advice I received felt personalized to me	3.13 (1.36)
The advice I received felt relevant to me	3.44 (1.31)
I followed the advice given to me in the resources	3.88 (0.72)
I completed the tasks in the 'Try it out' section in each of the resources	3.69 (1.01)
I will use the advice from the resources in the future	3.81 (0.98)

### Qualitative Feedback

Qualitative feedback was collected in two ways: through the posttrial feedback questionnaire and posttrial interviews. The feedback was mixed, with some people feeling very positive about the phenotypes and others feeling they were not as useful. One participant said the advice “was good” (Participant 9), and found it especially helpful to learn about “the reasons behind my eating when not hungry and the food I choose.” Another participant said:

all advice was spot on (…) I looked forward to reading them and I will miss them. More would be great (…) I found this the most helpful even more than the learning modules (…) [understanding] how my mind works in relation to food certainly explained a lot

Participant 10

Two more participants wrote, “Excellent advice & profiles” (Participant 11) and “I found the information encouraging to read” (Participant 14). Two participants felt more neutral about their experience, as one said, “it was all useful, except I haven’t lost very much weight” (Participant 13), and another said, “It was ok. To be honest I can’t recall much about it” (Participant 12). Others felt less positively about the phenotypes, saying the “quiz was not really helpful, advice was very vague and generalized (…) no specific tailored help” (Participant 2). Another participant said, “Too much emphasis on how I feel. I hate being constantly asked to think about how I feel. (…) I didn't really fit into any of the categories anyway” (Participant 4). Others said, “it just feels generic and not very personal” (Participant 7) and “[the profile] didn’t reflect me” (Participant 8).

Everyone who completed the posttrial survey was invited to participate in an interview, and 5 participants accepted. Participant 9 felt positively about the quiz and the Red Chilli profile she was matched with. She said the profile “was so accurate that it resonated” and “when I read the profile I was like (…) I know I'm an emotional eater (…) but actually, until I read that, it didn't make sense.” She talked about how the profile:

threw up things that you weren't aware of (…) you know what you should be doing. Why can't you do it? And I think that was the big question I really wanted to know the answer to (…) and then finding out that it's probably this and this (…) [that] was the most helpful thing.

Participant 10, who was also matched with the Red Chilli profile, said, “honestly, every week, I've looked forward to reading them [the advice]. And I'm just sorry they've stopped now.” She talked about how she used the learning from the profile to think:

How do I feel? Am I being, you know, not very kind to myself? (…) it put things into perspective for me rather than just saying I've ate this, I've drank that.

She mentioned how the advice was tailored to her needs, saying:

I think I've lost well over a stone (…) I don't have a bad diet, I don't think I just choose fast, convenient food when I'm stressed (…) I've just put more thought into it and the thought that not being cruel to yourself, being actually like, oh, OK, you can do this.

Moreover, she said:

I found the language to be absolutely, you know, spot on for me that it had got me down to a T (…) I'm hoping that it [the advice] will make me stop and think (…) to actually keep the weight off because that's the second challenge, isn't it?

Participant 6 was matched with the Yellow Saffron profile. She said:

quite often, diet or health things, they don't really allow for people who really love food in a foodie way (…) [the quiz] recognized that I was that sort of person (…) the advice was tailored to me (…) which actually I was really pleased with.

She also said:

I don't have to sort of feel defensive about it [being a foodie] (…) for dieting purposes, if you're in a particular profile, and they're not recognizing that in a positive way, so you get defensive and stressed (…) whereas this has recognized it in a positive way, and given me ways of thinking about food, how I like to live, support how I like to eat food (…) but at the same time, it said you could tweak this (…) I've probably been able to take a step back and view how I eat things in a slightly different way, but without feeling guilty about it.

In an interview with Participant 2, the participant said she did not feel her profile was the right fit and resonated with two profiles when shown all the profile descriptions. When asked for feedback, she was reviewing the core program rather than the phenotype advice and struggled to remember seeing the documents with the advice. Participant 7 opened 4 out of 7 documents with advice for her profile; however, due to a system error, she was not receiving any coaching on the program. Thus, in the interview, she primarily talked about her dissatisfaction with the course in general rather than about the phenotypes.

## Discussion

### Overview

The aim of this quasi-experimental study was to assess the impact of providing tailored weight loss advice on app engagements and weight loss within a digital weight loss program. Participants were patients enrolled in a weight management program offered by Oviva, a service provider for the NHS. Advice was tailored based on responses to a phenotypes quiz, which matched patients to 1 of 4 cognitive-behavioral profiles. The evidence-based, tailored advice was delivered weekly over email for 7 weeks. App engagement (eg, logging meals, reading lessons) was used as a proxy for program adherence.

### Effect on App Engagements

Patients who received phenotype-tailored advice generated significantly more app engagements over a 7-week period compared with a historical cohort of patients enrolled in the same program a year earlier and compared with patients who received an invitation to take the quiz but who did not click on the link (ie, nonresponders). There was a 61.5% increase in app engagements between the phenotypes group and the historical cohort and a 90.5% increase in engagements compared with the nonresponder group. There was a significant positive correlation between the number of documents opened and the number of app engagements generated.

This finding aligns with our hypothesis that receiving tailored advice would boost program adherence. Additionally, the “try it out” section in the documents, which included exercises such as logging hunger types in the app, likely contributed to increased engagement. This is unlikely to be driven purely by the instructions to use the app, as Oviva regularly encourages users to log activities in the app, and such elevated engagement was not observed in the comparison groups. It could be that the coupling between tailored advice and instructions to log in the app was particularly effective. This is an important finding, as adherence to digital programs, often measured by app engagement, can be challenging. Enhancing adherence to digital interventions is crucial because it directly improves health-related outcomes [49]. Therefore, strategies that increase adherence are highly valuable for the success of eHealth interventions [50-52].

The increased engagement observed in the phenotype group may have been influenced by self-selection bias, given the study’s quasi-experimental design. Because participants had to actively complete multiple steps (opening an email, clicking the questionnaire link, and completing it) to receive their phenotype “match,” it is possible that only the most motivated individuals chose to participate. This could, in turn, explain the higher engagement rates observed in this group. A randomized controlled trial is required to establish causality.

A randomized controlled trial could be feasibly integrated into digital weight management programs by randomizing eligible patients at the point of onboarding to either receive standard program content or phenotype-tailored advice in addition to the standard content. Randomization and content delivery could be automated within the app infrastructure, and outcomes (eg, app engagement, weight change) could be monitored through existing data pipelines. This design would allow for rigorous testing of the added benefit of phenotype-based personalization within a real-world setting, without changing or disrupting routine care.

Although engagement was higher in the phenotypes group, engagement levels declined week by week across all conditions. This pattern aligns with previous research on weight loss interventions [49-51] and Oviva’s internal data. One possible explanation is habituation—as users become familiar with the app’s features, the novelty may wear off, leading to reduced interaction. Additionally, some users may have consistent meal routines, logging the same foods frequently in the early weeks but gradually reducing this behavior over time. Given that meal tracking is typically the primary contributor to in-app engagement, a decline in logging frequency could naturally result in lower overall engagement levels.

There was a slight imbalance in IMD scores between the phenotype group and the historical cohort, raising the question of whether relative deprivation status influenced engagement. However, the analysis showed that IMD was not associated with engagement, and adding IMD to the model did not improve its explanatory power beyond the effect of group alone. Furthermore, the interaction term was not informative because of the shared variance between predictor variables. While this may suggest that phenotype-based personalization supported engagement across socioeconomic groups, the study was not powered to detect interaction effects, particularly given the unequal group sizes. Therefore, these null findings should be interpreted with caution.

### Effect on Weight Loss

We also predicted that phenotype-tailored weight loss advice would lead to greater reductions in body mass over a 7-week period. While the phenotypes group showed more weight loss (–2.23 kg) compared with the historical cohort (–1.60 kg) and nonresponders (–0.69 kg), these differences were not statistically significant. IMD was not associated with weight loss, and the interaction between group and IMD was also nonsignificant. Bayesian analysis further confirmed that a baseline intercept model was substantially better than models including group, IMD, or their interaction, indicating that none of these variables explained variation in weight loss. This lack of statistically significant differences in weight loss between the groups may be explained by the fact that applying tailored advice takes time both to master and to impact weight loss, and our 7-week trial may have been too short to observe significant effects. A longer-term follow-up with a larger sample may be worthwhile in future studies to more clearly elucidate whether phenotype-tailored advice can increase weight loss.

### Posttrial Feedback on the Phenotypes

Only a small proportion of participants (10.8%) provided posttrial feedback. While these results should be interpreted with caution, the phenotypes and tailored advice were generally well received. The 4 most endorsed statements were that the advice was clear and easy to understand, helpful, followed by participants, and likely to be used in the future.

Interviews revealed mixed opinions. Some patients found the phenotypes useful, as they helped them understand the underlying reasons for their maladaptive eating behaviors. Others, however, felt the advice was too generic. In the survey, the statement with the lowest endorsement was that the advice felt personalized. One possible explanation is that the term “personalized” created expectations of a high level of individualization that were ultimately unmet. Additionally, some participants reported that none of the profiles fully resonated with them. This is not unexpected, as the 4 profiles are not exhaustive or mutually exclusive. They were designed to provide general guidance on the cognitive and behavioral patterns most likely to hinder progress, and some individuals may relate to more than one profile or none at all.

A more hyperpersonalized approach, in which individual cognitive and behavioral dimensions are assessed separately and combined into tailored content, may improve both fit and impact. Such an approach is increasingly feasible through artificial intelligence–enabled systems. A recent study demonstrated that health advice can be tailored not only in terms of content but also in delivery, aligning with individuals’ natural tendencies and cognitive styles [52]. This dual approach to personalization opens new possibilities for future work.

Recent advances in large language models are enabling digital health companies to deliver increasingly personalized messages, insights, and content [53-55]. Incorporating phenotypes into retrieval-augmented generation systems [56] offers a promising opportunity to enhance both relevance and precision of tailored interventions [57]. By aligning content with users’ cognitive styles and behaviors, such systems may improve engagement and clinical outcomes [57-60]. Although this study did not directly examine artificial intelligence–driven personalization, combining cognitive-behavioral profiling with large language models represents a compelling avenue for future digital health innovation [60].

Another limitation relates to the delivery format. Some participants may have provided feedback on the core Oviva program rather than the phenotype-specific content (posttrial interviews confirmed at least 2 instances), making it unclear whether low personalization ratings reflected dissatisfaction with the advice or broader program frustrations. Delivering tailored advice through a separate medium (by email), while the main program was delivered in-app, may also have reduced coherence. Future studies would benefit from integrating tailored content directly into the digital intervention itself to create a more seamless, unified experience that could further improve engagement and outcomes.

### Strengths and Limitations

One strength of this study was testing phenotype-tailored advice on patients enrolled in an NHS-referred weight loss program, making our sample representative of UK patients seeking weight loss treatment and enhancing external validity. Additionally, by using an established program provider, we leveraged a well-developed app and core program, minimizing adherence issues due to app functionality, a common problem in prototype apps used in studies.

However, this study had limitations. The quasi-experimental design limits causal inferences. It is possible that because participants were not randomly assigned, only the most motivated individuals completed the phenotypes quiz, which could explain the higher engagement in this group. Without a randomized controlled trial or baseline motivation measurements, this alternative explanation remains possible.

Another limitation is that tailored content was delivered by email rather than integrated into the app, creating a disjointed experience that may have led participants to confuse the core program with the phenotype content. This separation could also have resulted in some participants missing the email content, leading to a fragmented experience. Finally, the phenotypes were based on data from 4 Oviva weight management programs, but the sample in this study was predominantly matched with the Red Chilli profile. Recruiting from a single program may have contributed to the observed differences in phenotype distribution. Such an uneven distribution might also indicate a need for further refinement of subgroups within profiles to better differentiate patient challenges.

Finally, weight was self-reported, which may have introduced bias (eg, social desirability, systematic underreporting), but self-reporting is standard practice in remotely delivered tier 2 programs and is accepted by the NHS for monitoring patient progress. In the program, participants track weight privately with their coach, with no penalties or incentives for weight change. In the study, phenotype-specific advice focused on cognition and behavior rather than weight outcomes, aligning with the app’s broader behavioral framing. Participants were not specifically prompted to report weight or emphasize weight loss, and were unaware it would be assessed for the trial. Importantly, any bias in weight reporting is unlikely to have affected the study’s primary aims, as analyses focused on differences across groups or time rather than absolute weight values.

### Conclusion

In conclusion, this study provided preliminary evidence that advice tailored to cognitive-behavioral phenotypes may increase program adherence, as indicated by app engagements. Although the quasi-experimental design limits causal conclusions, the initial data trends are promising. Despite limited posttrial feedback, the positive ratings suggest that further exploration of phenotype-tailored advice is warranted. To fully assess its impact on app engagement and weight loss, a randomized controlled trial with a larger sample and longer timeframe is needed.

## Appendix

Multimedia Appendix 1 All items in the phenotypes questionnaire, and additional app engagement analyses.

Multimedia Appendix 2 TREND checklist.

## Abbreviations

BF: Bayes Factor
DV: dependent variable
IMD: Index of Multiple Deprivation
NHS: National Health Service

## Data Availability

The datasets generated or analyzed during this study are not publicly available due to the potential risk of participant re-identification but are available from the corresponding author on reasonable request.
