# Supplementary material for: Personalizing a Weight Loss Program Using Cognitive-Behavioral Phenotypes to Improve Engagement and Weight Loss in Adults With Overweight or Obesity: Quasi-Experimental Study
Source: JMIR Form Res. 2025 Dec 1;9:e72645. doi: 10.2196/72645 (PMC12706443; doi:10.2196/72645)
Supplement: Multimedia Appendix 1 [file formative_v9i1e72645_app1.doc]

**Supplementary Materials**

**The phenotypes quiz**

The phenotypes questionnaire (‘quiz’), participants completed to be matched with one of four cognitive-behavioral profiles. All items rated on 1–5 Likert scale (Strongly Disagree to Strongly Agree)

1. When I feel sad, I often overeat
2. My eating feels like a ball rolling down a hill that just keeps going and going
3. I feel helpless about controlling my eating
4. I find myself eating despite negative consequences
5. I love food
6. I love thinking about food
7. I can get really excited thinking about food
8. Enjoying food is one of the most important pleasures in my life
9. Good news makes me feel overjoyed
10. I get a special thrill when I am praised for something I’ve done well
11. I always celebrate when I accomplish something important
12. I set goals for myself and keep track of my progress
13. I am able to accomplish goals I set for myself
14. If I make a resolution to change something, I pay a lot of attention to how I’m doing

Think about health-related goals you are trying to achieve (e.g. exercise more, eat more vegetables). Now, for each statement below, select the rating that best describes your thoughts and feelings about pursuing these health-related goals.

1. When I feel stressed by pursuing my health-related goals, I give up
2. When I feel discouraged by my health-related goals, I let my commitment slide
3. I put off pursuing health-related goals when I could be doing a more enjoyable task

**Additional analyses**

Effect of condition on total engagements, additional analyses

A series of independent t-tests were conducted, to assess whether the increase in app engagement seen in the phenotype group was driven by any particular type of app activity. The analyses revealed that all engagement types were higher in the phenotype group, than in the historical cohort, suggesting no individual activity type drove the observed effect.

**Table S1**
Comparison of different app activities for the phenotype group and historical cohort. SD in parentheses.

| **Engagement type** | **Phenotype group (n=148)** | **Historical cohort (n=241)** |  |
| --- | --- | --- | --- |
| Tracking (e.g. meals, weight, activity) | 181 (180) | 121 (148) | *t*(387)=3.60, *p*<.001 |
| Coach messages | 4 (6) | 2 (3) | *t*(387)=3.71, *p*<.001 |
| Pages of educational modules read | 66 (64) | 33 (48) | *t*(387)=5.68, *p*<.001 |
| Number of additional articles read | 7 (9) | 3 (6) | *t*(387)=4.78, *p*<.001 |
